# Potential Application of Lung Ultrasound in Children with Severe Uncontrolled Asthma: Preliminary Hypothesis Based on a Case Series

**DOI:** 10.3390/medicines9020011

**Published:** 2022-02-02

**Authors:** Cristina De Rose, Stefano Miceli Sopo, Piero Valentini, Rosa Morello, Daniele Biasucci, Danilo Buonsenso

**Affiliations:** 1 Department of Woman and Child Health and Public Health, Policlinic Gemelli Universitary Foundation IRCCS, Catholic University of Sacre Hearth, 00168 Rome, Italy; stefano.micelisopo@gmail.com (S.M.S.); piero.valentinti@policlinicogemelli.it (P.V.); rosa.morello@gmail.com (R.M.); danilo.buonsenso@gmail.com (D.B.); 2 Department of Anesthesia and Intensive Care, Fondazione Policlinico Universitario “A. Gemelli”, 00168 Rome, Italy; daniele.biasucci@policlinicogemelli.it; 3 Department of Basic Biotechnological Sciences, Intensive and Perioperative Clinics, Catholic University of Sacre Hearth, 00168 Rome, Italy; 4 Global Health Research Institute, Institute of Hygiene, Catholic University of Sacre Hearth, 00168 Rome, Italy

**Keywords:** lung ultrasound, LUS, children, asthma, personalized medicine

## Abstract

In recent years, lung ultrasound (LUS) has been increasingly used for the diagnosis of respiratory diseases in both adult and pediatric patients. However, asthma is a field in which the use of LUS is not yet well defined, or is in development. In the following case series, we describe clinical, laboratory, and radiological results, as well as detailed lung ultrasound findings of six children with asthma: some of them with acute asthma attack and with inadequately controlled allergic asthma or childhood asthma; others with acute asthma and allergic or infantile asthma adequately controlled by preventive therapy. Finally, we describe the clinical, laboratory, and imaging parameters of a child with severe allergic asthma in the absence of exacerbation. In these cases, albeit at different times, LUS played an important role in both the initial diagnostic process and follow-up. It also showed different ultrasound features depending on the severity of the individual asthma based on the type of asthmatic phenotype and control of it.

## 1. Introduction

Children frequently present with respiratory distress and concomitant wheezing as a manifestation of various respiratory diseases [1,2,3]. Pediatricians must determine whether the underlying pathophysiological process is related to acute airway infections or to asthma and/or wheezing for preschool children. The management of the aforementioned common conditions of childhood is dramatically different [4,5].

In the era of personalized medicine, a point-of-care tool capable of differentiating etiologies and/or guiding the management of individual children with respiratory diseases characterized by wheezing would prove useful for personalized assistance of these patients, starting from the first clinical evaluation.

The chronic inflammation underlying asthma guides the airway remodeling with consequent lung parenchymal structural changes whose severity is related to the asthmatic endotype-phenotype and to the therapeutic control of it [1,2,3,6]. The airway remodeling is a fundamental process in asthma, but difficult to measure [6].

To date, the gold standard for evaluating airways and lung parenchyma remodeling is the chest CT scan, which, for obvious reasons of radiation protection [7], cannot be routinely performed in children and adolescents. In fact, in children with asthma, chest CT is performed before classifying the patient as suffering from severe uncontrolled asthma to exclude the diagnosis of underlying lung diseases which can simulate the asthma [1,2,3].

In this context, in the pediatric population in which it is not possible to use routine radio invasive imaging, a non-invasive, easily usable, low-cost diagnostic tool would therefore be useful to use as a clinical biomarker to visualize, measure, and monitor signs of airway remodeling over time, and then evaluate their response to therapy.

In recent years, lung ultrasound (LUS) has been increasingly used for the diagnosis of respiratory diseases in both adult and pediatric patients [8,9,10]. However, asthma is a field in which the use of LUS is not yet well defined, or is in development. In fact, there are very few studies available to date in the literature [4,5].

In the following case series, we describe clinical, laboratory, and radiological results, as well as detailed lung ultrasound findings of five children with acute asthmatic attack, and a child with severe asthma in the absence of exacerbation. In particular, we have presented the cases of:

- 2 children with acute asthma attack and history of allergic asthma, but not adequately controlled with therapy;
- 1 child with acute asthma attack and history of childhood asthma not adequately controlled with therapy;
- 2 children with acute asthma attack and with history, respectively, of childhood asthma and allergic asthma adequately controlled with therapy; and finally,
- 1 child with severe asthma in the absence of exacerbation.

In these cases, albeit at different times, LUS played an important role in both the initial diagnostic process and follow-up. It also showed different results depending on the severity of the individual asthma based on the type of asthmatic phenotype and control of it.

Written informed consent was obtained from a parent or guardian before data collection. The study was approved by the Institutional Review Board and Ethic Committee (prot.36173/19 ID2729). All patients’ data were analyzed anonymously. The main settings were represented by the pediatric emergency department and pediatric ward. Ultrasound examinations were performed using a MyLab linear transducer at 12 MHz, and the small parts preset (EsaoteSpA, Genoa, Italy).

## 2. Case Descriptionts 

### 2.1. Case 1

Case 1 was an 8-year-old female. Her past medical history included an episode of bronchiolitis at the age of 6 months; frequent episodes of asthmatic bronchitis in preschool age, and subsequently recurrent bronchospasm. She also had a positive family history of atopy and asthma. At the age of six, she was diagnosed with allergic asthma (T2-atopic, non-eosinophilic phenotype [1,2,3,11,12]). However, no preventive therapy was prescribed and administered, despite the new diagnosis, and despite the girl continuing to have monthly episodes of bronchospasm, even severe. Therefore, her asthma was not controlled as demonstrated by an Asthma Control Test (ACT) of 19 points [13].

She came to our attention at the Pediatric Emergency Department (PED) for acute onset of severe respiratory distress with diffuse bronchospasms and moderate–severe acute asthmatic attack: she presented severely dyspneic and tachypneic, could not speak, was tachycardic, and had an oxygen saturation of 88%, resulting in high flow oxygen requirements. She was transferred from another hospital where she was diagnosed with acute asthma attack in the course of pneumonia on the basis of the chest X-ray that the colleagues from the other center had decided to perform to rule out any complications in consideration of the severity of the clinical picture. However, the girl was apyretic, and the inflammation indices were negative, as were the molecular and culture microbiological tests of the airways for both viruses and bacteria.

**Figure 1:**
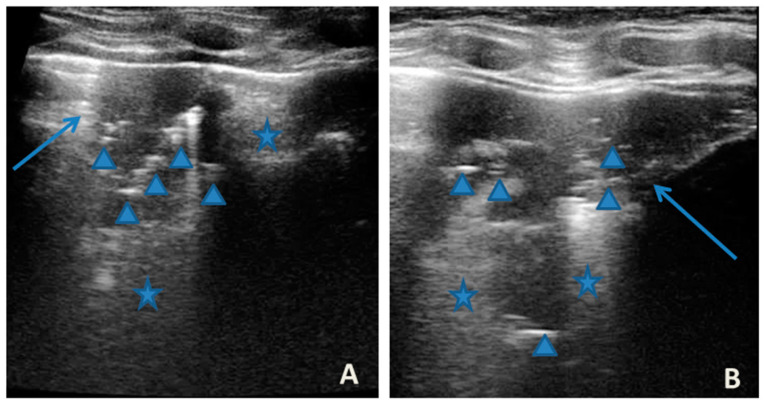
Grayscale lung ultrasound examination shows: (**A**): on the right anterior field, mainly in the mid-apical area, consolidation of atelectasis origin (arrow) with static air bronchograms (punctate), and parallel to each other (arrowheads), associated with sonographic interstitial syndrome (SIS), represented by coalescent vertical artefacts or B-lines (asterisks); (**B**): on the left paracardiac site, consolidation of about 2.5–3 cm of atelectasis nature (arrow) with static air bronchograms (punctate) (arrowheads), associated with sonographic interstitial syndrome (SIS), represented by coalescent vertical artefacts or B-lines (asterisks).

At our hospital, together with the clinical evaluation, we performed a lung ultrasound, which showed the presence of two areas of moderate lung atelectasis, in the right anterior mid-apical and in the left anterior paracardiac site, respectively, associated with sonographic interstitial syndrome (SIS) (Figure 1). LUS excluded the presence of inflammatory/infectious consolidations.

On the basis of all these data, the therapy of moderate–severe acute attack was set up with subsequent suspension of respiratory support after about a week of therapy. An adequate preventive therapy was also set based on the severity of the asthma, the phenotype, and the age of the patient [1,2,3]. 

The lung ultrasound follow-up showed complete resolution of the areas of atelectasis after 4 weeks from the start of background therapy, whereas the long vertical artifacts and irregularities of the pleural line persisted.

### 2.2. Case 2

Case 2 was a boy of 10 years old, with a medical history characterized by episodes of recurrent bronchospasms starting at 6 years of age, treated as needed with inhaled short-acting bronchodilators and several visits to the PED for severe acute asthmatic exacerbations. At the age of 8, allergic asthma was diagnosed (T2—atopic, hypereosinophilic phenotype [1,2,3,11,12]). Despite this, no preventive therapy was prescribed and administered. In fact, his asthma was not controlled, as shown by an ACT of 18 [13] calculated at the time of our evaluation.

**Figure 2:**
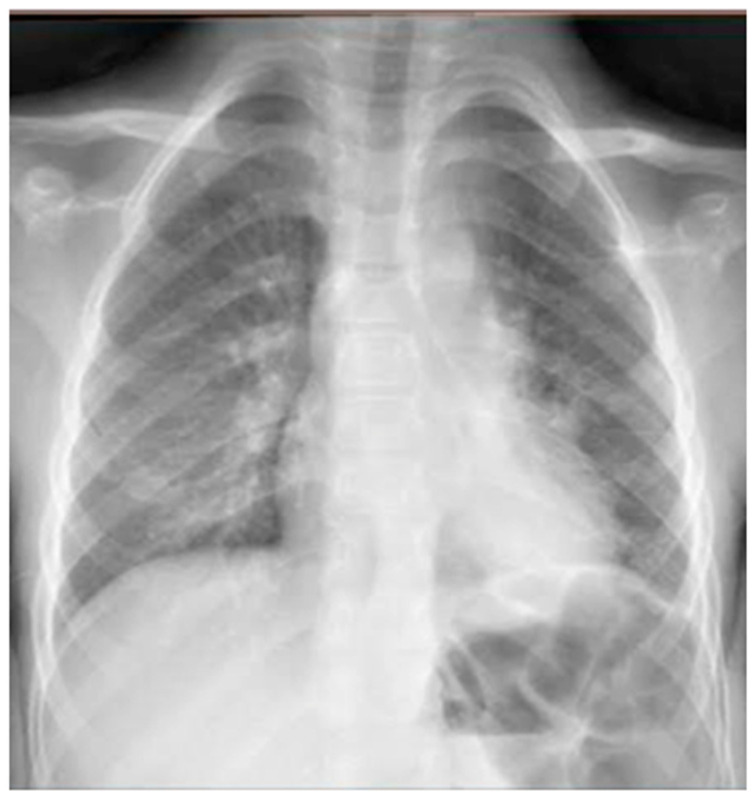
Chest radiography shows, on the left inferior lobe, non-specific areas of reduced transparency, associated with obliteration of the left lateral costophrenic sinus. It shows further subtle parenchymal hypodiaphania on the right mid-basal area.

He came to our attention at the PED for acute onset of severe respiratory distress with diffuse bronchospasms and moderate-to-severe acute asthmatic attack: he presented severely dyspneic and tachypneic, unable to speak, he was tachycardic, and he had an oxygen saturation of 88–90% with consequent oxygen requirement. A chest X-ray was performed in the pediatric emergency room to rule out respiratory complications in consideration of the severity of the severe clinical picture. The radiographic picture (Figure 2) was interpreted as an inflammatory type consolidation. However, the child was apyretic, and the inflammation indices were negative, as were the molecular and culture microbiological tests of the airways for both viruses and bacteria.

**Figure 3:**
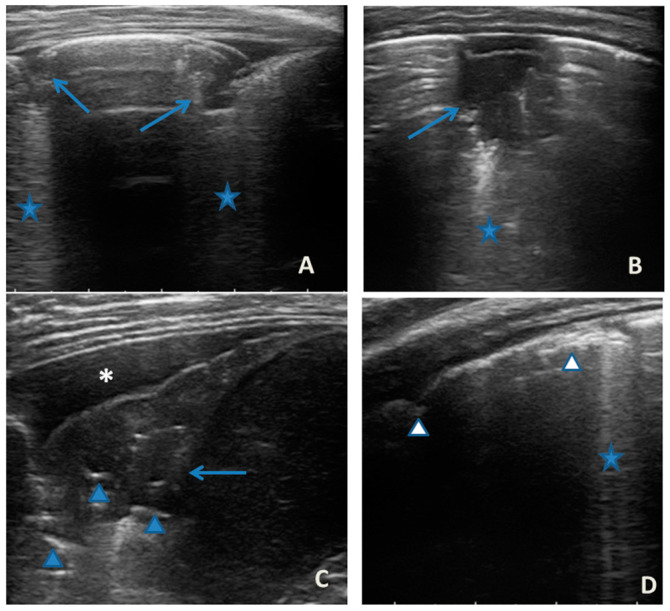
Grayscale lung ultrasound examination shows: an irregular pleural line in all fields explored; on the left anterior-lateral field, mainly in the apical area; (**A**) and on the left anterior retrocardiac field, mainly in the mid-apical area; (**B**) consolidations of an atelectatic nature (arrow), associated with sonographic interstitial syndrome (SIS), represented by coalescent vertical artefacts or B-lines and “white lung” areas (asterisks); (**C**) on the left postero-lateral field in the basal area, consolidation of about 3 cm of atelectasis nature (arrow) with static air bronchograms (punctate) (arrowheads), associated with reactive transudative pleural effusion (white asterisk); (**D**) on the right lateral fields, diffuse sonographic interstitial syndrome (SIS), characterized by irregularities of the pleural line (white arrowheads) and long confluent vertical artifacts (asterisks).

In any case, therapy for acute asthma attack and antibiotic therapy was set up, and he was admitted to the pediatric ward. Here, at the same time as the clinical evaluation, we performed LUS, which showed the presence of a diffuse SIS, and three areas of lung atelectasis, one of which—present in the left posterolateral basal area—was associated with mild reactive effusion (Figure 3). The LUS picture was highly positive, but excluded the presence of inflammatory/infectious consolidations.

On the basis of these data, the antibiotic therapy was therefore suspended, oxygen therapy was set at high flows in consideration of the presence of atelectasis, and the therapy of the moderate–severe acute attack was continued with subsequent suspension of respiratory support after about a week of therapy. Adequate preventive therapy [1,2,3] was also set up. The LUS follow-up showed complete resolution of the areas of atelectasis after 4 weeks from the start of preventive therapy, whereas long vertical artifacts and irregularities of the pleural line were still present.

### 2.3. Case 3 

Case 3 was a 2-year-old girl with a medical history of recurrent wheezing from 6 months of age, most of which present in the course of viral infections, and some episodes present even in the absence of respiratory infections. She had been prescribed inadequate preventive therapy, characterized only by antileukotriene agents, so she continued to have recurrent episodes of asthmatic bronchitis and nocturnal cough.

She came to our attention at the PED for acute onset of mild–moderate respiratory distress with diffuse bronchospasms and the presence of fever and rhinitis present for two days.

**Figure 4:**
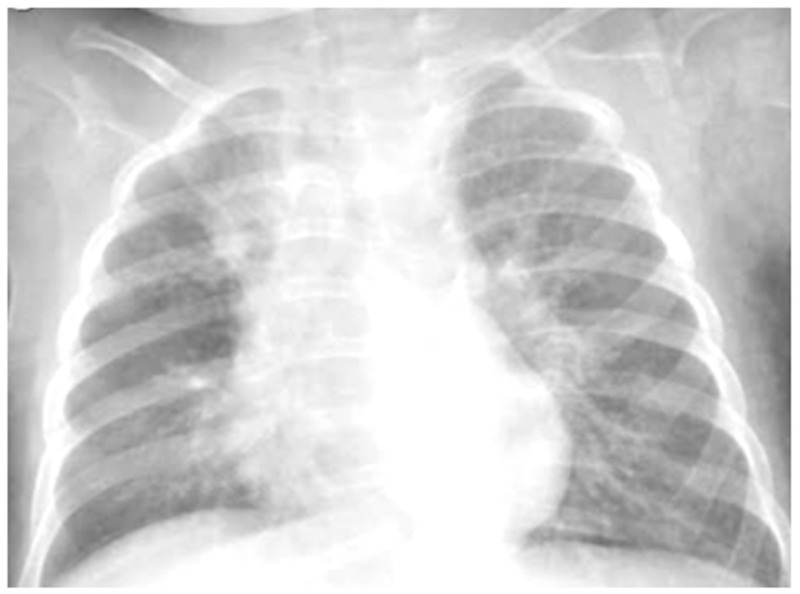
Chest radiography shows, on the right fields in the apical and basal areas, a non-specific area of reduced transparency. It also shows signs of interstitial engagement in the para-hilar position bilaterally.

**Figure 5:**
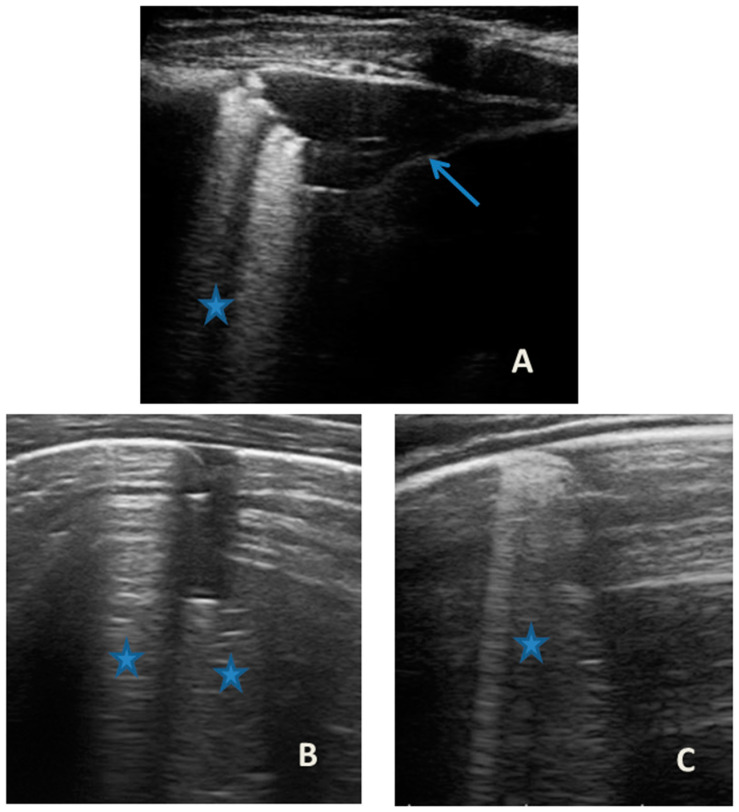
Grayscale lung ultrasound examination shows: an irregular pleural line in all fields explored; on the right anterior field, mainly in the medio-apical area, in the paracardiac area (**A**); and on the right anterior field in the basal area (**B**); consolidations of an atelectasis nature (arrow) poorly aerated, associated with sonographic interstitial syndrome (SIS), represented by coalescent vertical artefacts or B-lines and “white lung” areas (asterisks), which are also present in on the bilateral lateral fields (**C**).

She was moderately dyspneic, in good general condition, and with stable vital signs. She had a slight rise in the indexes of inflammation. At the PED, a chest X-ray was performed to exclude an infectious picture in consideration of the auscultator finding of reduced air penetration in the right hemi thorax. The radiographic picture (Figure 4) was interpreted as an inflammatory type consolidation. Therefore, therapy for acute asthma attack and antibiotic therapy was set up, and she was hospitalized in the pediatric ward. Here, at the same time as the clinical evaluation, we performed LUS, which showed the presence of a picture of SIS, and several areas of lung atelectasis, one of which involved the middle lobe—localized in the right anterior mid-apical area (paracardiac) (Figure 5). The LUS picture was highly positive, but excluded the presence of inflammatory/infectious consolidations. On the basis of these data, the antibiotic therapy was therefore suspended, high flow oxygen therapy was set in consideration of the presence of atelectasis areas, and therapy of the moderate–severe acute attack was continued with subsequent suspension of respiratory support after about a week of therapy. Furthermore, the microbiological investigations of the airways were positive for adenovirus. An adequate preventive therapy was set up [1,2,3]. LUS follow-up showed a complete resolution of the areas of atelectasis after 2 weeks from the start of preventive therapy, and of the SIS after about 4 weeks from the start of the therapy itself.

### 2.4. Case 4 

Case 4 was a 10-year-old boy, with a medical history characterized by hyperosinophilic allergic asthma (T2—atopic, hypereosinophilic phenotype [1,2,3,11,12]), well controlled by preventive therapy [1,2,3], with an ACT of 25 points [13].

He came to our attention at the PED for acute onset of acute mild asthmatic attack. The patient was generally stable, and had mild dyspnea, but vital signs were stable and he had no oxygen requirement. The indices of inflammation were negative; as were the cultural and molecular microbiological examinations of the airways.

**Figure 6:**
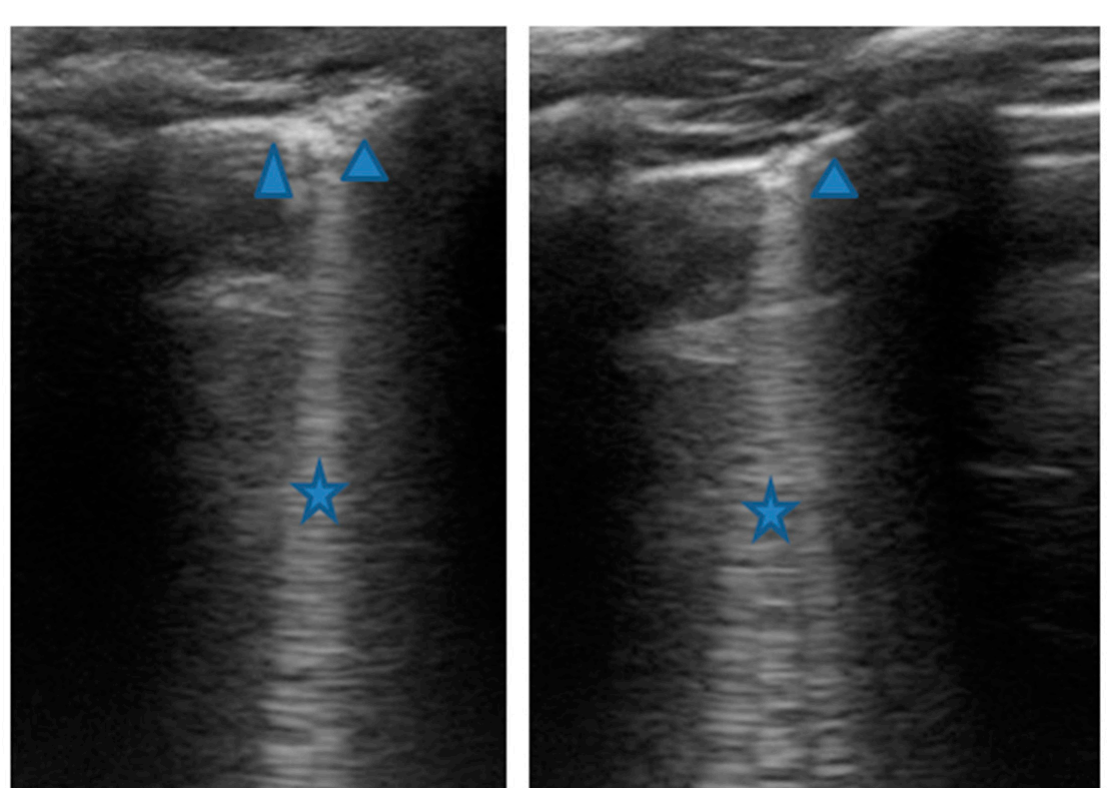
Grayscale lung ultrasound examination shows: sonographic interstitial syndrome (SIS), characterized by long confluent vertical artifacts (asterisks), associated with an irregular pleural line, and distributed over all the explored fields in an inhomogeneous way.

Acute asthmatic attack therapy was set up. Simultaneously with the first clinical evaluation, we performed LUS, which showed the presence of a diffuse SIS picture (Figure 6) in the absence of consolidations of both an atelectasis and inflammatory nature. We witnessed the resolution of symptoms after less than 48 of acute phase therapy, and the patient was discharged with the same preventive therapy he was doing. LUS follow-up showed persistence of sporadic single and non-confluent artifacts even after three weeks, with pleural line irregularities.

### 2.5. Case 5 

Case 5 was a 3-year-and-5-month-old girl with medical history of recurrent wheezing by viral infections. For a year, she had not had asthma exacerbations thanks to adequate preventive therapy [1,2,3].

**Figure 7:**
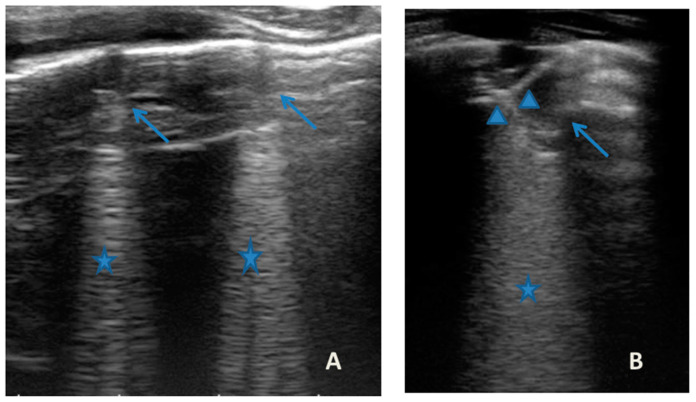
Grayscale lung ultrasound examination shows: (**A**): sub-centimeter consolidations less than 0.5 cm in size (arrows), associated with confluent artifacts (asterisks) in the left lateral mid-apical area; (**B**): a small consolidation of 0.5–1 cm (arrows), with elements of dynamic air bronchogram (arrowhead) (as per suspected inflammation) [10,14,15], associated with a circumscribed area of “white lung” (asterisks) in the right middle apical area; (**A**,**B**): irregularity of the pleural line. Picture compatible with acute inflammation of the small airways of viral origin [14,15].

She came to our attention at the PED for acute onset of asthmatic bronchitis, with mild respiratory distress, fever, and rhinitis present for three days. She was mildly dyspneic, in good general condition, and with stable vital signs. She did not need oxygen. Upon examination of the chest, she presented with diffuse bronchospasm, associated with rales spread throughout the lung area. She had a moderate rise in the inflammation indices. At the same time as the first clinical evaluation, we performed LUS, which showed a picture compatible with acute inflammation of the small airways (Figure 7). This picture was also confirmed by the microbiological data of positivity of the nasopharyngeal swab for RSV. Respiratory symptoms resolved after 72 acute phase therapy. The patient was discharged with the preventive therapy already in progress. LUS follow-up showed the complete resolution of the ultrasound picture after a total of 5 days.

### 2.6. Case 6 

**Figure 8:**
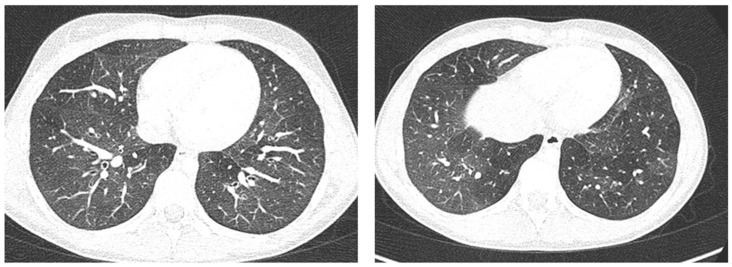
A representative coronal section from thoracic computed tomography (CT) scan reveals: thickening of the bronchial walls bilaterally, with filling of the bronchial lumen predominantly in the apical segment of the right upper lobe; picture of diffuse inhomogeneity of parenchymal density with diffuse and bilateral hypodense areas in relation to air trapping correlated to a picture of recurrent airway inflammation.

Case 6 was a 10-year-old girl with a medical history of allergic rhinoconjunctivitis recurring since childhood; her diagnosis of allergic asthma (T2-eosinophilic phenotype— “T2-high” [11,12]) was from 6 years of age. It has always been a severe phenotype despite adequate preventive therapy, so much as to require a progressive step-up of the therapy itself up to the maximum step [1,2,3]. Despite this, and the good adherence to therapy, she came to our attention at the pediatric allergy clinic of our hospital, reporting that she was still having recurrent symptoms, resorting daily to short-acting bronchodilators, and was also having nocturnal symptoms. She had an ACT of 20 points [13], and pathological control spirometry. Therefore, in consideration of the presence of severe eosinophilic T2 allergic asthma (T2-high), the patient was a candidate for the administration of a biological drug. However, before classifying the patient as such, a chest CT was performed to exclude the presence of other pathological respiratory conditions that could dissemble asthma (Figure 8).

**Figure 9:**
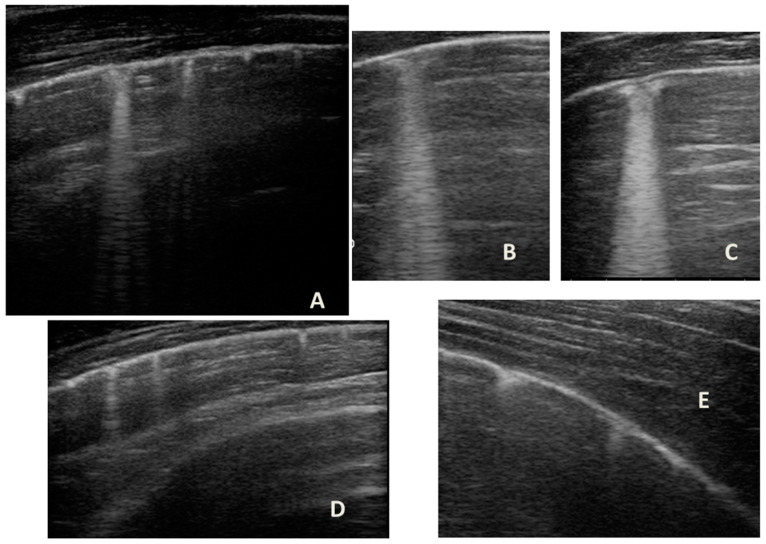
Grayscale lung ultrasound examination shows a picture of sonographic interstitial syndrome (SIS) represented by: (**A**): irregularity of the pleural line, short vertical artifacts, and long vertical artifacts unevenly distributed bilaterally; (**B**,**C**): irregularity of the pleural line, sub-centimeter consolidations associated with long confluent vertical artifacts located mainly in the right lung fields, mainly in the mid-apical area; (**D**,**E**): short vertical artifacts with pleural line irregularities unevenly distributed bilaterally.

LUS was also performed, which showed a picture compatible with the features detected on CT (Figure 9).

## 3. Discussion

Asthma is a complex heterogeneous disorder characterized by chronic inflammation of the airways, at the basis of which there are specific pathogenetic mechanisms (endotypes) that are responsible for a set of separate conditions (phenotypes) [11,12] that lead to a common clinical picture characterized with recurrent episodes of wheezing, shortness of breath, chest tightness, and cough [1,2,3,11,12]. The heterogeneity of the pathophysiology and clinical expression of asthma is becoming increasingly important in the era of “personalized” medicine, according to which there is now strong evidence that treatment should not be a “one-size-fits-all” approach [1,2,3,11,12], but should take into account the characteristics of each endotype-phenotype [11,12]. In fact, each phenotype has a different clinical severity, different comorbidities, and a different response to drugs used as preventive therapy for asthma [1,2,3,11,12].

Today, the diagnosis of asthma is based on the clinical and anamnestic history of respiratory symptoms that vary over time and in intensity; on the variable limitation of the expiratory air flow on physical examination; and on objective tests that demonstrate a variable obstruction air flow, such as spirometry [1,2,3]. Instead, the definition of the asthmatic phenotype is based on diagnostic tests with which it is possible to evaluate hyperactivity and inflammation of the airways [1,2,3,11,12].

Regarding the role of traditional imaging, asthma exacerbations are predominantly triggered by viral respiratory infections or inhalant allergens, and both conditions can trigger an acute inflammatory process that can overlap the underlying chronic inflammatory process with a different imaging pattern, depending on the severity and control of the asthmatic phenotype.

However, regarding chest X-ray, it does not provide useful information for the design of treatment plans for children with acute asthma [16], as in the vast majority of cases, it is completely negative, and in a minority, it is suggestive of atelectasis and/or thickening of the interstitium: elements that are often confused with a radiographic picture of pneumonia, with subsequent administration of antibiotic therapy not necessary to modify the outcome of acute asthmatic attack [16], just as it happened in the first three of our cases. The studies carried out so far are, in fact, all in agreement that chest X-ray is often not helpful in asthma [16].

On the contrary, chest CT remains the gold standard for evaluating both acute phase lung alterations [6], and to measure the airways and lung parenchyma remodeling in severe clinical pictures [6]. However, in the pediatric population, in the acute phase, it is not performed, as it does not change the type of treatment provided for the acute asthmatic attack in the absence of respiratory complications, and, in the same way, it cannot be routinely performed to evaluate airway remodeling due to radiation protection reasons [1,2,3,7]. 

As far as LUS is concerned, asthmatic pathology is a field in which the use of LUS is not yet well defined, or is in development [4,5].

In our cases (Table 1), particularly in the first three cases—children with asthma not controlled due to an inadequate preventive therapy for age, phenotype, and severity of the clinical picture [1,2]—LUS was highly positive (Figure 1, Figure 3 and Figure 5). It showed the presence of lung atelectasis, which resolved slowly after weeks of therapy of the acute attack and the beginning of adequate preventive therapy. On the contrary, in case 4 and 5—children with asthma well controlled by adequate preventive therapy—LUS was not highly positive (Figure 6 and Figure 7). In fact, it did not show large atelectasis, but a slight SIS in case 4, and a SIS suggestive of viral infection of the small airways in case 5 [14,15].

Furthermore, in some of these cases (both in controlled and uncontrolled cases by therapy), in particular those with the T2 phenotype, some of the ultrasound features (some elements of SIS in particular) found in the acute phase persisted even after months of preventive therapy, and in the stability phase.

Therefore, for all of these findings on LUS, we hypothesized that, in patients with uncontrolled asthma (cases 1–3), there could be an underlying important state of chronic inflammation—which is the cause of airway and lung parenchyma remodeling—to which the acute inflammation of the exacerbation is added, with the consequent formation of disventilatory areas of different entities that can appear ultrasonographically in the form of SIS or of atelectasis, depending on the severity [17]. So, to understand the ultrasound pattern found both in the acute phase and in the stability phase, and how this varies according to the characteristics of the underlying disease, it was important for each patient to know the phenotype, the level of clinical control, and whether the acute attack had been triggered by exposure to an allergen or a viral agent.

Several studies [10,14,15]—also performed in the pediatric population—show that LUS not only has a greater sensitivity and specificity than chest X-ray [10,14], but is also able to distinguish inflammatory/infectious consolidations from atelectasis [10,14], as well as distinguishing an inflammatory picture of viral origin from that of bacterial origin [14,15].

In our cases, LUS has allowed us to detect the aforementioned lesions, to adequately characterize them, and to follow them over time. In this way, we had the possibility to set up an adequate and personalized therapy for each patient both in the short-term and in the long-term, without using unnecessary therapies, such as antibiotic therapy.

Furthermore, evaluating some patients who had important ultrasound lesions (such as atelectasis of a certain entity, or diffuse sonographic interstitial syndrome), as in the first three cases, we wondered if some of these lesions may have already been present before the acute attack, and also in consideration of the finding of a positive ultrasound picture even in the stability phase, and after starting an adequate preventive therapy. Hence, there is a need to take into account the asthmatic phenotype and its severity for all patients, and also to evaluate the ultrasound patterns of patients in the stability phase in an outpatient setting, as in case 6. In the latter case—a patient with allergic asthma severe eosinophilic atopic type 2, but in a stability phase—the chest CT (Figure 8) showed signs of airway and lung parenchyma remodeling compatible with the patient’s severe clinical picture. LUS (Figure 9) showed a picture of diffuse short vertical artifacts, long confluent and non-confluent artifacts, and irregularities of the pleural line and sub-centimeter consolidations: lesions that, in the absence of acute respiratory pathology (as in our case), could be compatible with the remodeling pattern found on the chest CT.

Therefore, we asked ourselves whether it is possible to use LUS in the pediatric population in which it is not possible to routinely perform invasive radiological investigations [6,7] (1) as a clinical biomarker to visualize and monitor signs of airway remodeling over time, and in response to therapy; (2) to define a possible ultrasound pattern of asthma; and finally (3) in future clinical studies to characterize the pathophysiology of asthma, as well as other invasive tools that are used in studies/trials in the adult population (SPECT, PET.) [6].

Further studies are certainly needed to confirm our hypotheses, to validate a lung ultrasound pattern in asthmatic pathology that could help in the differential diagnosis of children with respiratory distress and wheezing, and, therefore, to standardize this new possible application for LUS. In the meantime, we suggest including LUS in the diagnostic and monitoring process of patients with acute and stable asthma because it can help in the diagnostic and monitoring process, as has happened for our cases.

## Figures and Tables

**Table 1:** Clinical results and lung ultrasound findings of case series.

	Case 1	Case 2	Case 3	Case 4	Case 5	Case 6
**Years and Gender**	8-year-old Female	10-year-old Male	2-year-old Female	10-year-old Male	3-year-and-5-month-old Female	10-year-old Female
**Asthmatic phenotype**	T2—atopic, non-eosinophilic	T2—atopic, hypereosinophilic	Recurrent wheezing/Childhood Asthma	T2—atopic, hypereosinophilic phenotype	Recurrent wheezing/Childhood Asthma	T2—atopic, eosinophilic phenotype—“T2-high”
**ACT**	19	18	/	25	/	20
**Clinical presentation**	Acute Asthmatic Attack -Severe dyspnea and tachypnea -Absence of language -Tachycardia -OSI: 88%	Acute Asthmatic Attack -Severe dyspnea and tachypnea -Absence of language -Tachycardia -OSI: 88–90%	Acute Asthmatic Attack/Asthamatic Bronchitis -Moderate dyspna -Presence of language -Fever and rhinitis -Stable vital signs	Acute Asthmatic Attack -Mild dyspnea -Presence of linguage -Stable vital signs	Acute Asthmatic Attack/Asthamatic Bronchitis -Mild dyspnea -Presence of language -Fever and rhinitis -Stable vital signs	Absence of acute state -Recurrent symptoms (cough, wheezing, fatigue)
**Severity grading of acute asthmatic attack**	Moderate–Severe	Moderate–Severe	Mild–Moderate	Mild	Mild	/
**LUS findings**	Two areas of moderate lung atelectasis, associated with SIS (Figure 1)	Diffuse SIS, and three areas of lung atelectasis, one of which associated with mild reactive effusion (Figure 3)	Diffuse SIS and several areas of lung atelectasis, one of which involved the middle lobe (Figure 5)	Diffuse SIS (Figure 6)	Acute inflammation of the small airways: sub-centimeter consolidations with elements of dynamic air bronchogram, associated with confluent artifacts/“white lung” (Figure 7)	SIS picture (Figure 9)

ACT: asthma control test; OSI: oxygen saturation index; SIS: sonographic interstitial syndrome.
